# Dynamic Functional Connectivity Patterns in Schizophrenia and the Relationship With Hallucinations

**DOI:** 10.3389/fpsyt.2020.00227

**Published:** 2020-03-31

**Authors:** Sarah Weber, Erik Johnsen, Rune A. Kroken, Else-Marie Løberg, Sevdalina Kandilarova, Drozdstoy Stoyanov, Kristiina Kompus, Kenneth Hugdahl

**Affiliations:** ^1^Department of Biological and Medical Psychology, University of Bergen, Bergen, Norway; ^2^Division of Psychiatry and NORMENT Centre of Excellence, Haukeland University Hospital, Bergen, Norway; ^3^Department of Clinical Medicine, University of Bergen, Bergen, Norway; ^4^Department of Addiction Medicine, Haukeland University Hospital, Bergen, Norway; ^5^Department of Clinical Psychology, University of Bergen, Bergen, Norway; ^6^Department of Psychiatry and Medical Psychology, Medical University of Plovdiv, Plovdiv, Bulgaria; ^7^Research Institute, Medical University of Plovdiv, Plovdiv, Bulgaria; ^8^Institute of Psychology, University of Tartu, Tartu, Estonia; ^9^Department of Radiology, Haukeland University Hospital, Bergen, Norway

**Keywords:** schizophrenia, psychosis, auditory verbal hallucinations, default mode network, neuroimaging, fMRI

## Abstract

There is a wealth of evidence showing aberrant functional connectivity (FC) in schizophrenia but with considerable variability in findings across studies. Dynamic FC is an extension of traditional static FC, in that such analyses allow for explorations of temporal changes in connectivity. Thereby they also provide more detailed information on connectivity abnormalities in psychiatric disorders such as schizophrenia. The current study investigated dynamic FC in a sample of 80 schizophrenia patients and 80 matched healthy control subjects, replicating previous findings of aberrant dwell times in specific FC states, and further supporting a role for default mode network (DMN) dysfunction. Furthermore, relationships with hallucinations, a core symptom of schizophrenia, were explored. Two measures of hallucinations were used, one measure of current hallucination severity assessed on the day of scanning, and one trait-measure where hallucinations were assessed repeatedly over the course of 1 year. Current hallucination severity did not show a significant relationship with dynamic FC. However, the trait-measure of hallucination proneness over 1 year showed a significant relationship with dynamic FC. Patients with high hallucination proneness spent less time in connectivity states characterized by strong anti-correlation between the DMN and task-positive networks. The findings support theoretical models of hallucinations which have proposed an instability of the DMN and impaired cognitive control in patients with hallucinations. Furthermore, the results point to hallucination proneness as a potential marker for identifying distinct subgroups of schizophrenia patients.

## Introduction

Schizophrenia (SZ) has been described as a “disconnection syndrome” (1), referring to aberrant interaction between critical areas of the brain. Neuroimaging studies have found evidence for altered resting state functional connectivity (FC) in schizophrenia patients compared to healthy control subjects, covering a range of different brain regions [see (2–5) for selected reviews]. These findings include hyper- as well as hypo-connectivity, with considerable variation in results across studies.

The inconsistencies in findings might partly stem from the fact that schizophrenia is characterized by a wide range of different symptoms which are associated with different biological underpinnings and distinct FC abnormalities. Hence, variability in sample compositions might affect the results of such studies. In particular, auditory verbal hallucinations, a key symptom of SZ, have repeatedly been linked to FC alterations. These alterations involve auditory and language regions, the default mode network (DMN), executive and cognitive control networks, and subcortical areas [see (6–8) for selected reviews].

The majority of fMRI studies that have investigated FC in schizophrenia and hallucinations, have employed methods of static connectivity, i.e. averaging FC across the scanning time. These methods work on the assumption that functional connectivity between brain regions does not change substantially across the duration of the scanning session which typically lasts 5–15 min. However, this assumption does not seem to hold true in the face of growing evidence for considerable fluctuations in fMRI FC across time (9, 10). Therefore, recent investigations of fMRI resting state FC have increasingly focused on dynamic, or time-varying, FC. These investigations aim to detect connectivity patterns that can be found across subjects and occur at different time points throughout the scanning period (11, 12). One of the main approaches for the investigation of dynamic FC is a sliding time window approach, where FC matrices are computed for consecutive portions of the scanning period. FC matrices of those windows can then be clustered based on similarities in the FC patterns in order to form a set of “connectivity states”. Each connectivity state is characterized by a distinct FC pattern across a variety of brain regions and the temporal pattern of those states over time describes the functional organization of the brain [e.g., (13)]. The occurrences of FC states within an fMRI session have been linked to concurrently measured EEG signals (14, 15) and to different task conditions (16). These findings suggest that FC states reflect fluctuations in neuronal activity and cognitive states, or modes of brain functioning. Furthermore, differences in dynamic FC also reflect more stable, inter-individual differences in cognition (17).

Measures of dynamic FC also show links with mental health disorders, such as schizophrenia, and have been successful in differentiating patient groups and healthy controls (18). Importantly, dynamic FC has been found to outperform static FC measures when classifying SZ patients (19, 20). Interestingly, classification was not significantly improved when adding static FC measures to dynamic ones. This suggests that SZ-related abnormalities in static FC are also captured in dynamic FC but not vice versa. SZ patients and healthy controls have been shown to differ on a number of variables related to dynamic FC. First, patients spend more time in states with overall weak FC between networks, and less time in states with strong FC between sensory networks (18, 21–23). Second, SZ show reduced “dynamism” in FC, reflected in a reduced number of distinct FC states and reduced switching between states (24). This reduction in dynamism was also specifically related to the severity of hallucinations. On the other hand, the amount of time spent in different FC states showed no relationship with a summary measure of positive symptoms which included hallucinations (21). Together, these results could point to a unique relationship between dynamic FC and hallucinations.

The current study sought to investigate resting state dynamic FC in SZ, with a particular focus on hallucinations. Given the relative scarcity of studies on dynamic FC in SZ, the first aim was to replicate previously reported differences between SZ and HC with respect to the length of time spent in different FC states (18, 21). In addition, links between dynamic FC states and hallucinations were explored, using two different measures of hallucinations. First, effects of current hallucination severity were investigated using a measure of hallucinations on the day of scanning. However, since previous research suggests that temporary symptom severity might not show a strong relationship with dynamic FC states (21), a second, trait measure of hallucinations was included. Hallucinations were assessed repeatedly over a 1-year period and a measure of hallucination proneness was established. This second measure reflects more stable trait differences between patients. Therefore, it could indicate distinct subgroups of SZ patients, which have been suggested in previous research based on lifetime history assessments of auditory hallucinations (25). Stable differences in hallucination proneness are likely to be reflected in brain functioning and may therefore also be related to dynamic FC, which reflects a general functional organization of the brain (26). Furthermore, dynamic FC has been shown to differentiate between patient groups with different diagnoses (18, 19) and symptom-based subgroups of patients (27). Therefore, we predicted differences in connectivity state dwell times between SZ patients and healthy controls, and between patients with low versus high hallucination proneness.

## Methods

### Subjects

fMRI data were collected from 84 patients with a schizophrenia spectrum disorder (SZ) according to the ICD-10 diagnostic manual (F20–F29: Schizophrenia, schizotypal, and delusional disorders) (28). Four data sets were excluded after pre-processing, due to head movements of more than one voxel size between volumes, resulting in 80 patient data sets. Eighty healthy control subjects (HC) were individually matched with the SZ patients based on gender (60 males per group) and age (± 3 years, except for six SZ-HC pairs with a mean difference of 8.12 years). The mean age was 30.96 years (SD = 11.91) for SZ and 30.86 years (SD = 11.13) for HC. The majority of patients used antipsychotic medication, of which all used second-generation antipsychotics, with some patients in addition using first-generation antipsychotics [defined daily dose (DDD) of antipsychotics M = 1.02, SD = 0.55]. Some patients also used anti-depressants (n=8), mood stabilizers (n=2), opioids (n=1), benzodiazepines (n=13), anticholinergic (n=4), anticonvulsant (n=4), or ADHD medication (n=1). Further information on the patient sample can be found in **Table 1**. All subjects gave written informed consent to take part in the study prior to participation.

**Table 1:** Demographic data for the whole sample of SZ patients, and for the 68 SZ patients from the Bergen site, split into non-hallucinators and hallucinators. Positive and Negative Syndrome Scale (PANSS) data are based on assessments on the day of fMRI scanning. A significant group difference between hallucinators and non-hallucinators was found for PANSS total (p =.041).

	all patients (n = 80)	non-hallucinators (n = 19)	hallucinators (n = 49)
Age	30.96 (11.91)	33.42 (11.38)	28.88 (11.15)
Gender (m/f)	59/21	13/6	36/13
PANSS P3	2.69 (1.63)	1.05 (0.23)	3.33 (1.49)
PANSS positive	16.23 (5.20)	13.53 (5.06)	16.73 (5.18)
PANSS negative	15.86 (5.00)	13.53 (5.17)	16.15 (4.63)
PANSS general	33.42 (8.99)	28.47 (8.71)	34.77 (8.85)
PANSS total	65.51 (16.39)	55.53 (16.24)	67.65 (15.09)
Medication (DDD)	1.02 (0.55)	0.96 (0.41)	1.08 (0.63)
Duration of illness	4.83 (7.71)	3.19 (5.47)	4.03 (7.15)

### Data Acquisition

#### Neuroimaging Data

3T MR data were acquired at the Haukeland University Hospital in Bergen, Norway (68 patients and 80 HC), and at the Medical University of Plovdiv, Bulgaria (12 patients). In the course of the study, the MR scanner at the Bergen site was upgraded from a GE Signa HDx to GE Discovery MR750. All data acquired at the Plovdiv site were acquired on a GE Discovery MR750w, using the same MR parameters as at the Bergen site. The scanner version was included as a regressor variable of no interest in all analyses (i.e., Bergen pre-upgrade, Bergen post-upgrade, Plovdiv). The study protocol was approved by the Regional Committee for Medical Research Ethics in Western Norway (REK Vest) (REK #2016/800) and by the ethical authorities at the Medical University of Plovdiv, and conducted according to the Declaration of Helsinki.

fMRI resting state data were collected during a 5.33-min eyes-closed scan. One hundred sixty whole brain volumes were acquired, with 30 slices with a 0.5 mm gap (voxel size 1.72×1.72×3 mm) with the following parameters: repetition time (TR)/echo time (TE)/flip angle (FA)/field of view (FOV) 2000ms/30ms/90°/220mm. In addition, a structural T1-weighted image was acquired (7.42 min) using a 3D SPGR sequence with the following parameters: TR/TE/FA/FOV 7.78ms/2.94ms/14°/256mm (post-upgrade: 6.9ms/3.0ms/14°/256mm), isotropic voxel size of 1mm^3^.

#### Clinical Data

Hallucination severity was assessed with the P3 item of the Positive and Negative Syndrome Scale [PANSS; (29)]. The PANSS P3 item assesses hallucinations in different modalities and has a particular focus on auditory hallucinations and hearing voices (30), since these are the most common type of hallucination in psychotic patients (31, 32). Therefore, the PANSS P3 item is a measure of hallucination severity in general but the way that the interview questions are organized reflects auditory hallucinations to a greater degree than other sensory modalities. All PANSS raters were trained and certified and satisfactory inter-rater reliability was documented. For all patients, PANSS data were collected on the day of fMRI scanning. For the 12 patients from the Plovdiv site, this was the only PANSS assessment, but for the 68 patients who were scanned at the Bergen site as part of a 1-year study, additional PANSS data were acquired during up to seven additional visits to the clinic (baseline, week 1, week 3, week 6, month 3, month 6, month 9, month 12). The mean number of visits per patient was M = 5.81 (SD = 2.04), with most patients being followed for at least 6 months (mean last visit number M = 6.51, SD = 1.74). **Supplementary Table S1** provides an overview of the distributions of visits per patient. fMRI data were typically acquired at one of the first visits (visit 1, 2, or 3 in 75% of cases), but in order to account for any potential effects of the time point of scanning, the visit number was included as a regressor variable of no interest when comparing patient subgroups.

### Data Preprocessing and Analyses

Data pre-processing was conducted with the SPM12 software package (https://www.fil.ion.ucl.ac.uk/spm/). This included realignment of functional volumes for head motion correction, coregistration of the T1 structural image to the mean functional image, normalization of functional data into MNI (Montreal Neurological Institute) space, resampling to a voxel size of 4x4x4 mm, and smoothing with a Gaussian kernel of 8 mm FWHM.

#### Independent Component Analysis (ICA)

Pre-processed fMRI data were further analyzed in the SPM Group ICA of fMRI Toolbox (GIFT v3.0b http://trendscenter.org/software/gift/), using the Toolbox default parameter settings. Initially, a two-step data reduction procedure was followed. First, all data sets underwent a subject-specific Principal Component Analysis (PCA) which estimated 150 components. Second, all subjects’ reduced data sets were concatenated and underwent a PCA which estimated 100 components on the group level. Subsequently, group-level spatial ICA was performed on the PCA output, identifying 100 functional components equivalent to Allen et al. (13) and Damaraju et al. (21). The Infomax algorithm was used for component estimation. Subject-specific component maps and time courses were obtained from the group-level components using the GICA back reconstruction method. Components were then evaluated and excluded as artifact components if they showed a peak in white matter or cerebrospinal fluid, or if the majority of the power in the Fourier frequency spectrum of the component’s time course was above 0.1 Hz (33, 34). Using component spatial maps from previous research (13) as templates, 45 components were finally identified as functional components belonging to one of eight functional networks: subcortical (four ICs: putamen, caudate, 2x thalamus), auditory (two ICs), somatomotor (eight ICs), visual (nine ICs), insula (one IC), fronto-parietal task-positive networks (eight ICs), Default Mode Network (eight ICs), and language (five ICs).

#### Dynamic Functional Connectivity Analysis

Dynamic functional connectivity analyses were conducted with the GIFT Dynamic FNC Toolbox (v1.0a) equivalent to previous research (13, 21). In a first step, subjects’ component time courses were detrended and despiked. A fifth-order Butterworth low-pass filter with a high frequency cutoff of 0.15 Hz was applied. Six realignment parameters obtained during head motion correction were regressed out from the time courses. For the dynamic FC analysis, overlapping time windows of 40 s (20 TRs) were taken from the scanning time in steps of 2 s (1 TR) and convolved with a Gaussian of sigma = 3 TRs in order to de-weigh volumes at the beginning and end of the windows. For each window, FC was estimated in the form of a regularized inverse covariance matrix using the Toolbox graphical LASSO method with an additional L1 norm constraint. Finally, covariance estimates were Fisher-Z-transformed. For each subject, variance associated with the scanner version was removed from the windowed FC. In order to identify FC states that reoccured across time and across subjects, the windowed FC matrices were subjected to the GIFT k-means clustering procedure. In a first step, a subset of windows (i.e. “exemplars”) was selected for each subject, representing those FC matrices with maximal variability in FC. From those windows, the optimal number of clusters (k) was determined by the toolbox using the elbow criterion, defined as the ratio of within-cluster distances to between-cluster distances. The resulting k cluster centroids were used as templates for clustering all windows’ FC matrices of all subjects.

#### Group Comparisons of Dynamic Functional Connectivity

The primary measures of interest were the state dwell times, indicating for each state the average length of the single time periods that subjects stayed in that FC state, before switching to another state. To test for differences between SZ and HC, a MANOVA was conducted with each state’s dwell time as dependent variables, and SZ versus HC as an independent variable. To investigate the relationship between dwell times and hallucinations, two different measures were employed. First, the continuous P3 score on the day of fMRI scanning was used as a measure of current hallucination severity. Second, P3 assessment over the course of 1 year was used as a trait measure of hallucination proneness. This resulted in a non-hallucinator group (n=19) whose P3 score was never higher than two in the 1-year period (or alternatively one score of three compensated for by only scores of one on all other assessments), and in a hallucinator group (n = 49) whose P3 score was three or higher in at least one assessment during the 1-year period (unless compensated by only scores of one on all other assessments). Two MANCOVAs were conducted, one for each of the two hallucination measures as an independent variable, and dwell times in the different FC states as dependent variables. Age, gender, MRI visit number, and type of medication were included as regressor variables of no interest, since hallucinators and non-hallucinators were not matched with respect to these variables.

## Results

### Dynamic Functional Connectivity States at the Whole-Group Level

**Figure 1:**
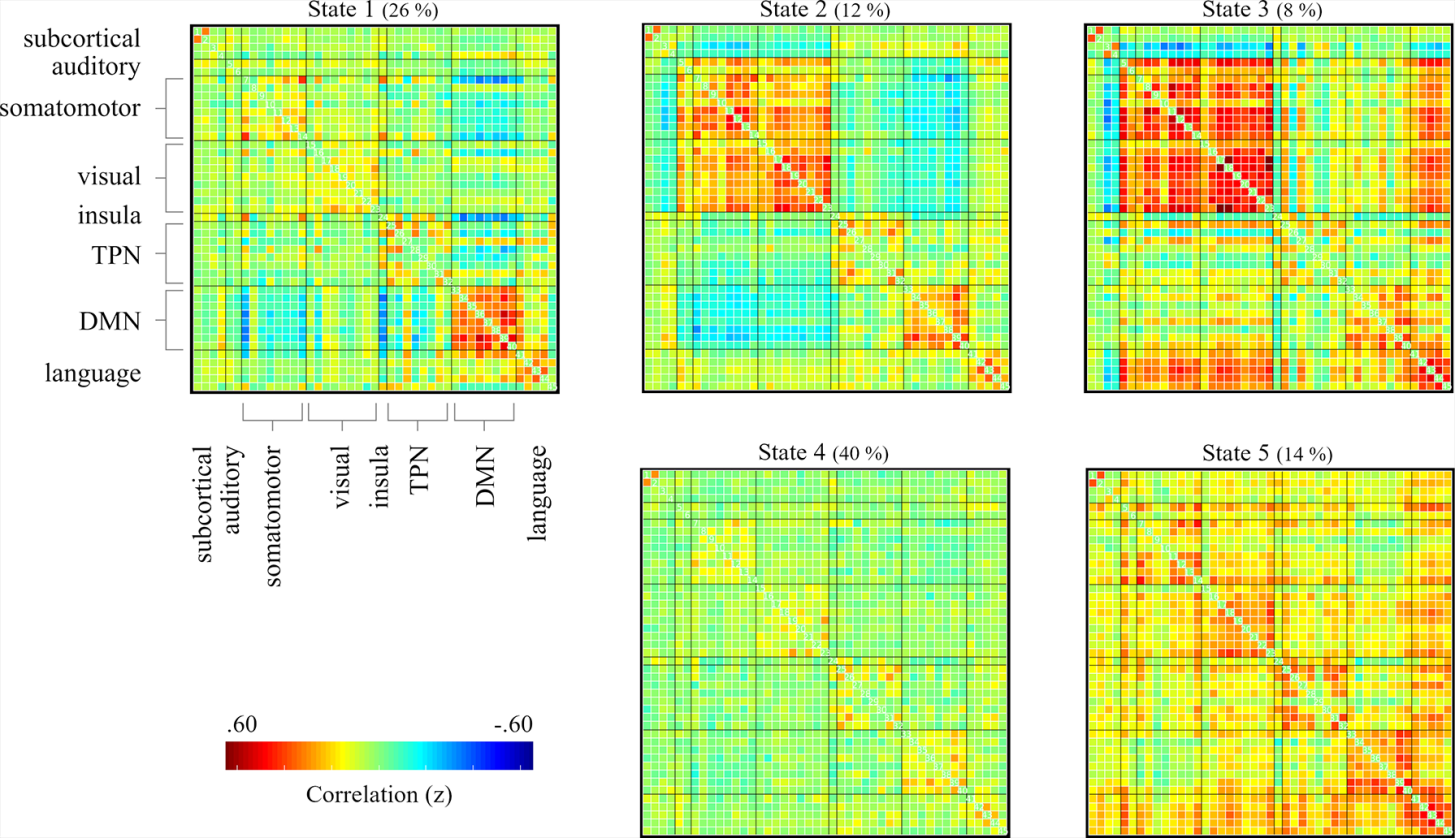
FC matrices of the dynamic FC states identified by k-means clustering on the whole-group level (80 SZ and 80 HC). Medians of cluster centroids are displayed. In brackets are the percentages of occurrence of the five states across the scanning period. The labelling of networks that is shown for state 1, is identical for all states. FC, functional connectivity; SZ, Schizophrenia; HC, healthy control.

K-means clustering identified five distinct connectivity states based on the windowed FC matrices (**Figure 1**). State 1 was characterized by strong positive FC within the DMN, i.e. between the different components of the DMN, and negative FC between the DMN and Task Positive Networks (TPN), especially the insula. State 2 was characterized by strong positive FC within and between sensory networks and negative FC of those networks with the DMN and TPN. State 3 was similar to State 2 but FC of sensory networks was even stronger and the negative FC with DMN and TPN was absent. Instead, sensory networks showed negative FC with subcortical networks. State 4 was characterized by overall weak FC across networks, which was positive within networks and negative between networks. State 5 was characterized by mostly positive FC which was strong within networks, in particular the DMN and language network.

### Differences in Dynamic FC Between SZ Patients and HC

**Figure 2:**
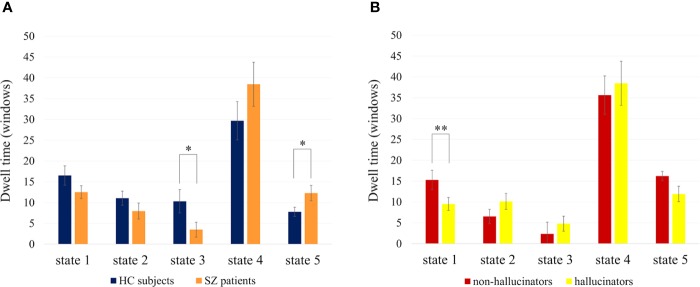
Mean dwell times (and SEM) in the different FC states per group. **(A)** Shows the comparison of HC subjects and SZ patients. **(B)** Shows the comparison of hallucinators and non-hallucinators. Dwell times are given in number of windows (1 window = 1 TR = 2s). Significant differences in dwell times are marked with * for *p* < .05 and ** for *p* < .01. FC, functional connectivity; TR, repetition time.

Two of the five FC states showed a significant difference in dwell times between SZ and HC (**Figure 2A**). SZ had significantly increased dwell time compared to HC in State 5, *F*(1,158) = 4.43, *p* =.037, but significantly reduced dwell time in State 3, *F*(1,158) = 5.01, *p*=.027. With respect to the occurrences of the two states in each group, fewer SZ than HC visited State 3 (n = 11 vs n = 23), whereas there was no difference for State 5 (n = 37 vs n = 36). There was no significant difference between SZ and HC in the number of distinct time periods in State 3 and State 5 (*p* =.299 and *p* =.578).

### Relationships Between Dynamic FC and Hallucinators

Current hallucination severity, assessed with the PANSS P3 score on the day of fMRI scanning, was not significantly related to dwell times in the different FC states (all *p* > .26). However, trait hallucination proneness over a 1-year period showed a significant relationship with state dwell times (**Figure 2B**). Compared to the non-hallucinator group, the hallucinator group had significantly reduced dwell times in State 1, *F*(1,26)=12.48, *p*=.002. There was no significant difference in the number of distinct time periods in State 1 (*p*=.452). Within the hallucinator group, the severity of hallucinations, measured as the mean P3 across 1 year, did not have a significant effect on dwell times (all *p* > .508, with *p*=.521 for state 1). In order to ensure that the results were specific to hallucinations and not driven by general symptom severity, the MANCOVA was repeated twice, once corrected for the positive PANSS score across visits and once corrected for the total PANSS score across visits, and the results remained substantially the same (changes in effect size from partial η^2^ =.32 to η^2^ =.26 and η^2^ =.29, respectively). Furthermore, neither the positive PANSS subscale nor the total PANSS score were significantly related to dwell times in State 1 (*p*=.130 and *p*=.151).

## Discussion

This study investigated dynamic FC in SZ patients compared with HC subjects and explored relationships with hallucinations, one of the key symptoms of SZ. On the whole-group level, dynamic FC analyses revealed five FC states which reoccurred over time and across subjects and which were characterized by distinct FC patterns across a variety of functional networks. These FC states were highly similar to those found in previous research using the same methods (13, 21). Dwell times in different states showed relationships with SZ diagnosis as well as hallucinations.

### Dynamic FC Differences Between SZ Patients and HC Subjects

While all FC states occurred in the SZ as well as in the HC group, the two groups differed with respect to the length of time periods spent in two out of the five states. Firstly, SZ had significantly longer dwell times than HC in states that were characterized by positive FC within and between networks and particularly strong FC within the DMN and the language network (State 5). The DMN has repeatedly been reported to show abnormal FC patterns in SZ, with the majority of reviews concluding predominantly increased FC within the DMN (3, 35, 36). This finding has been suggested to reflect thought disturbances in relation to an increased internal focus of cognitive processing in SZ patients (37, 38). The fact that SZ patients in the current study were not more likely to reach states with high within-DMN connectivity but stayed in these states significantly longer than HC, might indicate that they become “stuck” in these states of increased internal processing focus. Furthermore, those states were also characterized by overall positive FC across all brain regions, which suggests a weak segregation between different functional networks and could be related to impairments in differentiating between internal versus external thought contents (36).

The results also showed a significant difference between SZ and HC in dwell times in State 3. That is, HC stayed longer in states with strong connectivity within and between sensory networks and strong anti-correlation with subcortical areas, as has been reported previously (18, 21, 22). This FC state has been suggested to reflect a state of low alertness or drowsiness (13, 21). Shorter dwell times for SZ compared to HC could simply reflect higher levels of arousal or anxiety in patients, which would make them less likely to be in a state of relaxation or drowsiness. Even though this explanation is speculative, it is supported by the fact that more HC than SZ patients ever reached this state at all.

Overall, the differences between SZ and HC with respect to dwell times in the different FC states largely replicate previous findings (21, 23), even though some group differences did not pass the significance threshold in the current study (e.g., increased dwell time for SZ compared to HC in states with overall weak connectivity between all networks). Given the smaller sample size in the current study, this might be related to lower statistical power or it could indicate that some state differences are more generalizable across different sample compositions than others.

### Dynamic FC Differences Between Hallucinators and Non-Hallucinators

The second aim of the study was to explore the relationship between dynamic FC and hallucinations within the SZ sample. As expected (21), the time spent in different FC states was not significantly related to a measure of current hallucination severity on the day of fMRI scanning. However, assessing hallucinations as a trait variable across a 1-year period and differentiating between patients with high versus low hallucination proneness in that period, showed that those two subgroups of patients differed in dynamic FC. Specifically, hallucinators spent less time than non-hallucinators in states with strong anti-correlation between the DMN and task-positive networks (TPN).

Reduced DMN-TPN anti-correlation has previously been found in SZ patients in studies using static FC. These findings have been interpreted in the light of impaired differentiation between internal and external focus in cognitive processing (35, 36). These impairments might be particularly relevant for hallucinations, which constitute an internally generated stimulus which is attributed to an external source [cf. (39, 40)]. Abnormalities in DMN connectivity have also been linked to hallucinations directly (6). A recently proposed model of auditory hallucinations centers around aberrant DMN connectivity, alongside auditory cortex abnormalities (41). Specifically, it was suggested that a reduced anti-correlation between the DMN and the central executive network causes a collapse of states with an internal processing focus. The resulting DMN withdrawal then leads to a state of increased focus on auditory processing and consequently to the experience of hallucinations. The fact that hallucinators in the current study could not uphold DMN-TPN anti-correlation states for as long as non-hallucinators, might reflect the hypothesized collapse of internal processing states, which are maintained by DMN-TPN anti-correlation. Therefore, the findings are also in line with a previously proposed hallucination-related instability of the DMN (42) and a dysfunction of the cognitive control network (43).

### Hallucinations as a Potential Marker for Subgroups of SZ

The current study suggests that hallucinations could be an indicator for identifying distinct subgroups of SZ patients. Importantly though, relationships of hallucinations with dynamic FC were only present for trait hallucination proneness but not for current hallucination severity. This indicates that the general vulnerability to experience hallucinations is reflected in functional brain organization and that this vulnerability is detectable even in periods where hallucination severity is low (as indicated by patients with high hallucination proneness but low current hallucination severity). Within the hallucination-prone subgroup, there was no effect of hallucination severity over time, which suggests that differences in dynamic FC reflect a categorical vulnerability to experience hallucinations.

Differences between hallucinators and non-hallucinators persisted when correcting for total symptom severity and positive symptoms. Furthermore, neither total symptom severity nor positive symptoms were related to state dwell times. This points to a unique relationship of hallucinations with dynamic FC patterns and further supports the potential value of hallucinations as a marker for differentiating SZ subgroups. The measure of hallucination proneness over a 1-year period might be related to assessments of lifetime history of hallucinations, which has previously been shown to discriminate between SZ subgroups in a classification study (25). In fact, classification accuracy was higher for the hallucinators versus non-hallucinators subgroups of patients than for the SZ versus HC groups. This led the authors to the conclusion that hallucination-based patient subgroups might be a more useful entity than traditional diagnosis groups. In the current study, dynamic FC was susceptible to both, SZ diagnosis as well as hallucination proneness, but with stronger relationships with for hallucination proneness. Interestingly, the FC states that showed effects of SZ diagnosis and the states that showed hallucination effects, were both characterized by strong FC within the DMN. It is therefore possible that the two effects are related, with states of DMN-TPN anti-correlation (State 1) potentially compensating for DMN hyper-connectivity (State 5). That is, SZ patients generally spend more time in states with DMN hyper-connectivity, which has been associated with misattributions of internal thought contents to external sources (36–38). However, in non-hallucinators, states of DMN-TPN anti-correlation modulate DMN functioning, which might make these misattributions less likely and hence protect against hallucinations.

### Limitations and Directions for Future Research

Despite the potential of dynamic FC as a method for investigations of naturally occurring fluctuations in brain functioning, some limitations should be considered when interpreting dynamic FC findings. First, while dynamic FC corresponds to fluctuations in cognitive states (14, 16) and stable inter-individual cognitive differences (17), the interpretations of the single FC states with respect to underlying cognitive processes are still largely unclear. Even though FC patterns can be interpreted based on the knowledge about the functional networks involved, future research should investigate direct links between dynamic FC states and cognitive states, for example using task-based fMRI paradigms. With respect to hallucinations, it would be interesting to apply dynamic FC to fMRI data from symptom capture designs. This would allow an investigation of the states that are associated with time periods in which patients are known to experience hallucinations, or time periods that precede or succeed hallucinations.

Second, dynamic FC analyses require a number of choices with respect to different analysis parameters, such as window size, and the selection of functional networks. While it has been shown that some of these choices do not significantly affect the results (13, 21), the current study nonetheless used previously validated analysis parameters in order to enhance comparability between studies and offer a replication of previous findings (21). However, relationships between dynamic FC and hallucinations, which were based on a relatively small sample in the current study, should be subjected to further testing and replication.

A further critical point concerns the fact that fMRI data in the current study were collected on different scanners. While this is very common, in particular when studying clinical populations, great care should be taken when combining fMRI data from multiple sources, in order to avoid effects of sampling bias and scanner-related measurement parameters (44). In the present study, scanner-related variance was removed from the data so that all reported results are corrected for potential site- or scanner-related effects.

The current study did not assess the degree to which patients experienced hallucinations during fMRI scanning. Therefore, it is impossible to determine if and how potentially occurring hallucinations during scanning may have affected the results. This is particularly true for the analysis on current hallucination severity, which was assessed on the day of scanning and might therefore be related to hallucination frequency during scanning. Since this analysis did not show any significant effect on dynamic FC, it is difficult to speculate about a potential role of ongoing hallucinations in the current findings.

The current study used the PANSS P3 item as a measure of hallucination severity. Therefore, it is not possible to disentangle the effects of hallucinations in different sensory modalities. However, given the focus in the PANSS P3 interview situation on auditory hallucinations (30), and the relative prevalence of auditory hallucinations compared to other types of hallucinations (31, 32), it is likely that the results primarily reflect effects of auditory hallucinations. The reported hallucination-related differences were centered around FC abnormalities in the DMN and TPN and were interpreted in the context of theoretical models that were developed to explain auditory verbal hallucinations (41, 43). However, since the DMN and TPN are involved in general cognitive functioning, independent of a particular sensory modality, it is conceivable that their dysfunction does not only play a role in auditory hallucinations but in hallucinations generally. Future studies that assess different modalities of hallucinations separately, should explore to which degree they share underlying mechanisms, and to which degree modality-dependent differences exist.

## Data Availability Statement

The raw data supporting the conclusions of this article will be made available by the authors, without undue reservation, to any qualified researcher.

## Ethics Statement

The studies involving human participants were reviewed and approved by the Regional Committee for Medical Research Ethics in Western Norway (REK-Vest 2016/800); Medical University of Plovdiv. The patients/participants provided their written informed consent to participate in this study.

## Author Contributions

SW contributed data analysis and interpretation of the data and wrote the first draft of the manuscript. EJ contributed conception and design of the study. RK, E-ML, SK, and DS contributed to the data collection. KK organized the database. KH contributed to the conception and design of the study and interpretation of the data. All authors contributed to manuscript revision.

## Funding

The present research was funded from an ERC Advanced Grant #693124 and a Helse-Vest grant #912045 to KH and grants from the Research Council of Norway (RCN) #213727, and the Western Norway Regional Health Authority #911820 and #911679 to EJ.

## Conflict of Interest

KH owns shares in the NordicNeuroLab Inc. company, which produces some of the add-on equipment used during data acquisition.

The remaining authors declare that the research was conducted in the absence of any commercial or financial relationships that could be construed as a potential conflict of interest.
