# Supplementary material for: Dynamic Functional Connectivity Patterns in Schizophrenia and the Relationship With Hallucinations
Source: Front Psychiatry. 2020 Mar 31;11:227. doi: 10.3389/fpsyt.2020.00227 (PMC7145395; doi:10.3389/fpsyt.2020.00227)
Supplement: Supplementary file 1 [file DataSheet_1.pdf]

Supplementary Table S1. Distributions of clinic visits for which PANSS data were acquired for each patient from the Bergen site.

	Baseline	week 1	week 3	week 6	month 3	month 6	month 9	month 12
P01	x	x	x	x	x	x	x	x
P02	x	x	x	x	x	x	x	
P03	x				x			
P04	x	x	x	x	x	x	x	x
P05	x	x	x	x	x	x	x	x
P06	x	x	x	x	x	x	x	
P07	x	x	x	x	x	x	x	x
P08	x	x	x	x	x			
P09	x			x	x			
P10	x	x		x				
P11	x				x	x		x
P12	x				x			x
P13	x	x	x	x				
P14					x	x		
P15	x	x	x	x	x	x	x	x
P16	x	x	x	x	x	x	x	x
P17	x	x	x	x	x			
P18	x	x	x	x	x	x		
P19	x	x	x	x	x	x	x	x
P20	x	x	x	x	x	x	x	x
P21	x	x	x					
P22	x			x				
P23	x	x	x	x	x			
P24	x	x	x	x	x		x	
P25	x	x	x					
P26	x	x	x	x	x	x	x	x
P27	x	x	x	x	x	x	x	x
P28	x	x	x	x	x	x	x	x
P29	x	x	x	x	x	x	x	x
P30	x				x	x		
P31	x	x	x	x				
P32	x	x	x	x				
P33	x	x	x	x	x			
P34	x	x	x	x	x	x	x	x
P35	x	x	x	x				
P36	x	x	x	x	x	x		
P37	x	x	x	x	x	x	x	x
P38	x	x	x	x				
P39	x	x	x				x	
P40	x	x	x	x	x		x	x
P41	x	x	x				x	x
P42	x	x	x	x		x		x

P43	x	x	x	x	x	x	x	x
P44	x	x	x					
P45	x	x	x	x	x	x		
P46	x	x	x	x	x	x		
P47	x	x	x	x				
P48	x	x	x	x	x	x	x	x
P49	x	x	x	x				
P50	x	x	x	x		x		
P51	x	x	x	x	x	x	x	x
P52	x	x	x	x	x		x	x
P53	x	x	x	x	x		x	x
P54	x	x	x	x	x	x	x	x
P55	x				x	x		x
P56	x	x	x	x	x	x	x	x
P57	x	x	x	x	x	x	x	x
P58	x	x	x	x	x		x	
P59	x	x	x	x	x	x	x	x
P60	x	x	x	x	x	x	x	x
P61	x	x	x	x	x	x	x	x
P62	x					x		x
P63	x	x	x					
P64	x	x	x	x				
P65	x	x	x	x				x
P66	x	x	x	x		x	x	
P67	x	x	x	x	x	x	x	
P68	x	x	x	x	x	x		
